# Abortion in Domesticated Animals

**Published:** 1897-04

**Authors:** Charles F. Dawson

**Affiliations:** Veterinary Inspector, Bureau of Animal Industry


### ABORTION IN DOMESTICATED ANIMALS.

By Charles F. Dawson,
VETERINARY INSPECTOR, BUREAU OF ANIMAL INDUSTRY.

(Concluded from page 127.)

The earliest accounts of infectious abortion in the domesticated animals were written as far back as the third century before the Christian era; from that early time down to the present the disease has existed in a more or less severe form, often amounting almost to a scourge in stock-raising countries, and has received the attention of the ablest veterinarians of all times.

Professor Nocard made an extended study of the subject, and his report to the French Department of Agriculture shows him to be a strong advocate of the theory of the infectious nature of the disease. Another French veterinarian, Professor Lebat, showed his belief in the infection-theory by treating a flock of aborting ewes by means of local applications of antiseptics to the vulvæ of all the pregnant ones.[1]

In the report of the United States Department of Agriculture for 1883 Dr. D. E. Salmon wrote as follows: "This disease, which evidently depends upon some form of contagion for its causation, has been estimated to produce an annual loss in the State of New York alone of several millions of dollars. If, as seems likely from our general knowledge of the contagion, the germs of this disease are first scattered upon the stall-floors and upon the ground where the cattle run, to be taken into the system by soiled food, by the dust which rises and floats in the air, or in some similar way, then a thorough and continuous disinfection of the stables and runs should have a very marked effect in controlling it. In one rather

[1] Recueil de Méd. Vétérinaire, September 15, 1896.

large and plainly infected herd I have put this idea into practice with the happiest results, the disinfectant being a 1 per cent. solution of sulphuric acid."

Judging from the numerous articles upon the subject which have appeared in the *Agricultural Journal* of Cape Colony, Africa, the disease must be quite common in that country. In one of these articles[1] Mr. Hutcheon, the Colonial veterinary surgeon, says in discussing the probability of the infectious nature of the disease: "Practically, however, all cases of abortion should be treated as if it were a contagious disease, for while many individual cases may occur which do not affect others, it is an undoubted fact that abortion very often becomes infectious, and, if precautions to prevent its spread are not taken, serious losses are certain to follow."

An important paper upon the subject of infectious abortion was written by Dr. W. L. Williams,[2] who was appointed by the department to investigate the disease. Dr. Williams tabulates ten cases in which he gives the details of certain experiments undertaken to prove the infectiousness of material removed from the vaginæ of aborting mares. His experiments fail generally, however, to prove what he wished, he having caused only one animal to abort under conditions which could leave little doubt that the abortion was caused by the introduction of utero-vaginal discharges into the vulva of another pregnant mare. This mare aborted thirty-one days after. The period of incubation here seems to be a great drawback to the theory he advocates. It is difficult to understand why the material he introduced into the vulva did not manifest its pathogenic property in a shorter space of time. Dr. Williams states that the weather was unprecedentedly warm during the experimentation. This being the case, and as no mention is made of any extra precautions taken to prevent the multiplication of saprophytic bacteria in the material used, could we not reason that the hypothetical organism of abortion is one which quickly loses its virulence when associated with rapidly growing saprophytes in material rich in organic matter? In the one case where he produced abortion mention is made that the material was immediately transferred from the uterus and vagina to the vulva of the experimental mare. This one successful experiment in ten is, however, offset by a failure to produce abortion by the use of some of the same material under the same conditions in another mare. Could not this failure

[1] Agricultural Journal, vol. iii. No. 13, p. 326.

[2] Sixth and Seventh Annual Reports of Bureau of Animal Industry, Department of Agriculture, pp. 449–456.

be explained on the theory of lack of susceptibility? Could not his failures, generally, be explained on the same theory? All the animals used, with one exception, and the author does not say whether this exception was the one in which abortion was induced, were from a herd of unbroken Texas mares free from abortion. The excepted mare had been in the State of Illinois, where the experiments were carried on, for some time previous to the introduction of the others, and had been in foal twice by a native horse. Might not the results have been different if the experimental animals had been selected at random from native Illinois stock?

Bernes[1] reports an outbreak of abortion in a herd of cows which he successfully combated by measures based on the assumption that the disease was caused by infection. By removing the affected and healthy animals to separate buildings and thoroughly cleansing and disinfecting the building in which the outbreak occurred no more abortions took place. All the cows were served by the same bull, and one of them had a vaginal discharge a few days after being served. Indications point to the probability in this outbreak of infection having been carried by the bull. The number of exposed animals was quite large—fifty-two in number—while the reported number of abortions was only nine.

We have recorded many instances where this disease, like many others, raged for a while and then disappeared spontaneously. Would not this outbreak have conformed to this rule regardless of the sanitary measures adopted? It seems plausible that others should have aborted, did we not know that an outbreak of abortion is somewhat self-limited in character. It is very probable that the specific agent is one which loses part of its virulence from contact with the blood and utero-vaginal secretions. That the glands in the mucous membrane secrete a fluid which has germicidal properties seems plausible, from the fact that others, as well as myself, have failed to get any growth in the ordinary bacterial culture-media after placing in it material taken from the uteri of animals in health, to which the male had not recently had access.

Dr. T. J. Turner,[2] in his article upon infectious abortion in mares, records the results of certain experiments made upon pregnant mares to produce abortion. On June 6th Dr. Turner introduced into the system (method not given) of a pregnant mare near term a culture made from diseased fœtal membranes from a case of abortion. The mare foaled the following night. Dr. Turner says:

[1] Journal of Com. Path. and Therapeutics, 1891, vol. iv. p. 167.
[2] The Veterinary Journal, vol. xxxvii. 1893.

"On June 29th the foal showed signs of joint-trouble in the right knee, and on July 1st the hock-joint was as large as a man's head. Thus, from this experiment, almost just begun, we might say, Do we produce the disease in a colt that when born was apparently in health, and that, too, after the inoculation had only been introduced a few hours? Another mare, a dun, inoculated with a culture from the blood of Biddy Mac's colt (culture used in the other experiment was from fœtal membrane of same case. C. F. D.) on the 20th day of June, gave birth to a dead foal. This was an abortion, as evidenced by the diseased placenta. Hence we see that from these two inoculations with cultures we have produced both the diseases —abortion and joint-trouble. The germs causing these two diseases are the same, as shown under the microscope. That these two maladies are one and the same disease, but differently manifested, there is no doubt."

In the absence of more details concerning the experiments made by Dr. Turner we cannot accept his work as conclusive evidence that the case of abortion which took place, apparently the result of the introduction into the system of a pregnant mare of a culture of bacteria from the blood of a dead fœtus, was due to a specific organism. It is possible that the severe arthritis from which the offspring of the first experimental mare suffered may, as he claims, be due to the presence in the affected joints of an organism identical with the one he claims was the cause of abortion in the other mare. His statement claiming such identity, however, must be sustained by more evidence than a similarity in the morphology of the organisms.

Dr. F. L. Kilborne[1] investigated an outbreak of abortion in the mares of a large stud near Franklin, Pa. He says in his report: "No history of any introduction of the infection from without could be obtained beyond the fact that mares were daily being received at the stock-farm from various parts of the State, to be bred to the stallions of the stud. But as far as could be ascertained no mare had been received from any stable in which abortions had previously occurred. The veterinarian in charge of the stables had taken the usual precaution to remove the aborting mares as soon as discovered and to disinfect the stall. Such mares were disinfected per vaginam by repeated injections of a diluted solution of corrosive sublimate, 1 part to 10,000 of water, and the external organs, tail, and hind limbs sponged over with a strong

[1] Bulletin No. 3, Bureau of Animal Industry, Department of Agriculture.

solution, 1 part to 1500 of water. The stable had also been repeatedly fumigated with burning sulphur. Fluid extract of black-haw (*Viburnum prunifolium*) in half-ounce doses once daily had been given the pregnant mares during the month preceding February 5th. In this outbreak the first case of abortion occurred on December 2d, the birth being premature by 109 days. Up to the time of Dr. Kilborne's first visit—February 5th—ten cases had occurred, ranging in prematurity from 23 to 195 days. In addition to the sanitary measures already practised by the resident veterinarian, Dr. Kilborne advised a more thorough disinfection of the stalls with a 2 per cent. solution of sulphuric acid. The abortions ceased from January 29th to February 15th, when another outbreak occurred, lasting till March 4th, six mares having aborted. It was then decided to take additional precautionary measures to check the outbreak. All pregnant mares were removed to another building. The floors of the stalls were thoroughly cleansed and disinfected with a 2 per cent. solution of sulphuric acid twice a week. The external genitals and tail were washed daily with bichloride of mercury solution. Intravaginal injections of the mercury solution caused the animals to strain, and were discontinued. Half the mares were given once daily one-half ounce of the fluid extract of black-haw in addition to the sanitary measures taken. The remaining half of the mares received in the feed one heaping teaspoonful of the following powder-mixture twice daily for two weeks, when it was omitted for four or five days: chlorate of potassium, phosphate of sodium, of each one pound; and sulphate of quinia, three ounces. This treatment and the rigid sanitary measures seem to have quelled the outbreak, no more abortions occurring till in the fall, when three of the mares brought in from the pasture and stalled in a small barn with several others aborted. This new outbreak was checked by a repetition of the treatment and disinfection measures employed in the former outbreaks.

A bacterial culture was made from the vaginal mucous membrane of one of the cases by Dr. Kilborne, and experiments were projected by Dr. Theobald Smith to determine the presence in it of a bacterium which had pathogenic properties. The culture obtained proved upon examination to contain only a single species, a short motile bacillus. Cultures in peptone-bouillon were made from the original culture, and were injected into the vagina of a mare nine months pregnant, with the result that in twenty-four hours the mare had an abundant purulent vaginal discharge. The discharge ceased in two days. This experiment was spoiled by the mare developing a severe case of influenza. She foaled in about a week after the injection, probably the result of the attack of influenza.

Intravaginal injections in several pregnant cows did not give any positive results, with the exception of a temporary vaginal discharge.

Intravenous injections of this bacillus into hogs caused only a temporary anorexia. Inoculations made into rabbits of this bacillus proved fatal in three out of four cases, the animals dying of a disease simulating hog-cholera in rabbits. Dr. Smith inclines to the belief that on account of its pathogenic properties and its cultural characters that the organism obtained from the case of abortion in a mare is closely related to the bacillus of hog-cholera. He says,[1] in discussing its peculiarities: "Another fact of interest in connection with this pathogenic bacillus is its close resemblance to the hog-cholera bacillus . . . and if the bacillus in question had been sent me as having come from swine I should not have hesitated to regard it as a hog-cholera bacillus of rather feeble virulence, and possessing some slight differential characters from the virulent form."

In order to determine whether this bacillus is present in the genital passages of healthy pregnant and non-pregnant mares, Dr. V. A. Moore made a series of observations upon the vaginal secretions of five healthy mares, one of which was pregnant. These observations are recorded in Dr. Smith's report. Dr. Moore isolated eleven species of bacteria of the following genera: two species of non-motile bacilli; four species of micrococci; and five species of streptococci. Neither of the species of bacilli had characters which would show they were identical with the bacillus of which Dr. Smith wrote.

Recognizing the importance of having an intimate acquaintance with the normal bacterial flora of the genital passages as a prerequisite for the future investigation of infectious abortion in animals, I, at the suggestion of Dr. V. A. Moore, undertook a series of observations to determine those forms normally present in the vaginæ of cows.[2]

My method for collecting the vaginal secretions was as follows: a cotton applicator, made by twisting absorbent cotton round the roughened end of a piece of stout copper wire, was thrust into a test-tube through its cotton-wool stopper. The whole was placed

[1] Bulletin No. 3, Bureau of Animal Industry, Department of Agriculture, 1893, p. 58.

[2] The description of the biologic characters and the drawings of these organisms are reserved for a future communication.

in a sterilizer kept at 140° C. for one hour. This proved an excellent means of transporting the secretions free from contamination. When the sample was to be taken the external genitals and under the surface of the tail were thoroughly washed with a 2½ per cent. solution of carbolic acid, followed by a bath of sterilized distilled water and dried by means of sterilized cotton-wool. An assistant held the tail to one side, the labia were separated, and a speculum, anointed with carbolized vaseline, was introduced and held in place by an assistant. By opening the speculum the interior of the vagina and the os uteri could be plainly seen, and the applicator could be applied to the parts and bring away secretions it had absorbed during contact. Upon reaching the laboratory the applicator was removed from the tube, and twirled around in a flask of about 50 c.c. of melted agar. By this means any bacteria which were removed from the vagina in the meshes of the cotton were washed out. The infected agar was then poured into a double dish ten inches in diameter and similar to the ordinary Petri pattern. By the use of this size dish ample room was given for the development of a large number of different organisms from a single inoculation. From the large number of species from bacteria which these observations show are present in the bovine vagina, and the infrequency of the occurrence of the same species in different observations, it would seem that the flora of the bovine vagina is either very extensive or that the presence of such large numbers of species is accidental.

If we admit that they gain entrance to the vagina from the ventral surfaces of the tail and from the soiled vulvar surfaces, it would seem that most of them came from the feces.

Upon reflection we find that the rumen is a most admirable bacterial incubator because of the fact that in this portion of the alimentary canal the very conditions exist which are necessary for the development of bacteria—*i. e.*, warmth, moisture, and an alkaline reaction. Flourens has shown that fluids pass with facility from the rumen into the other three stomach divisions, and might we not, therefore, expect to find a very large number of species of bacteria by this means washed from the stomach into the intestine and appearing in the feces of these animals?

It is only in the abomasum, fourth or true stomach, that we find conditions that are inhibitive to the multiplication of swine-bacteria. When a ruminant swallows water some of it passes without much delay into all four of the stomach-divisions. It is probable that many of the bacteria are thus washed into the fourth stomach, and that their sojourn there is of short duration, many of them passing through the pylorus with the chyme into the intestine, where conditions are more favorable for their multiplication.

It would, therefore, seem that the number of species to be found in the bovine vagina is limited in a measure by the number of species swallowed with the food.

The observations were made upon perfectly healthy cows which were not known to have been in contact with a male for some time previous to my operations.

By the method given above forty-five species of bacteria of the following genera were studied: twenty-one species of bacilli, thirteen of which were motile, two of them being spore-producers, and eight of which were non-motile, two of these being spore-producers; seven species of micrococci, seven species of diplococci, eight species of staphylococci, and one streptococcus.

